# The Way Forward Is the Way Back

**DOI:** 10.3201/eid1303.000000

**Published:** 2007-03

**Authors:** Polyxeni Potter

**Affiliations:** * Centers for Disease Control and Prevention, Atlanta, Georgia, USA

**Keywords:** Francisco Roa, contemporary realism, still life, Spanish realism, tuberculosis, science-humanities connection, art and science, art commentary, about the cover

**Figure:**
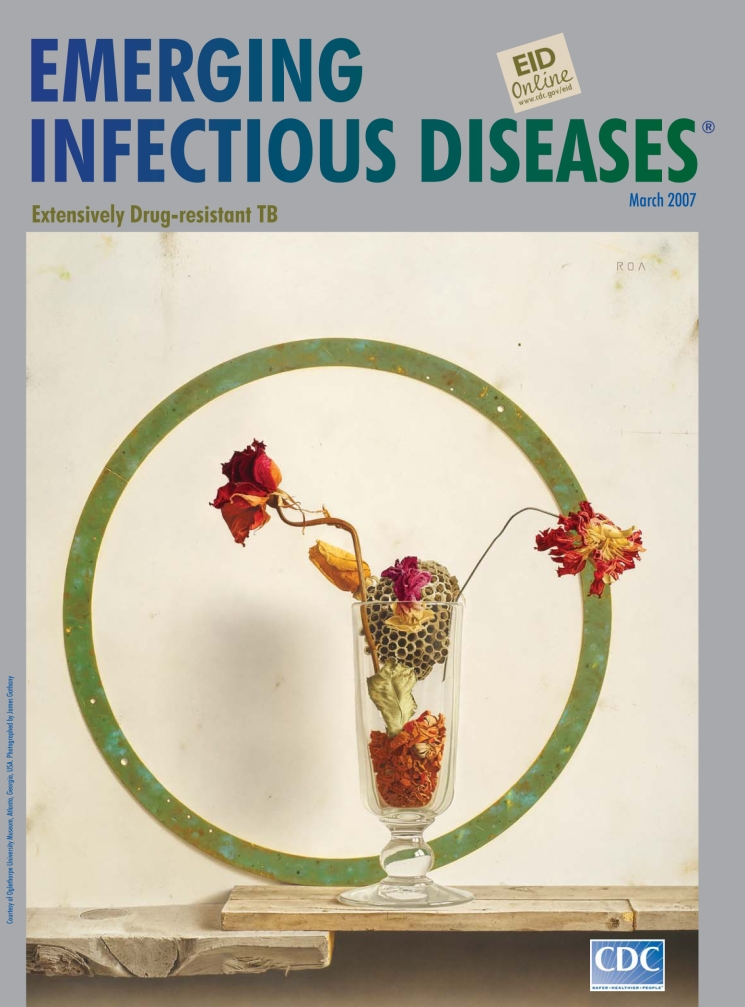
**Francisco Roa (b. 1963). Sands Flowers (1994).** Oil on panel (45 cm x 36 cm). Oglethorpe University Museum, Atlanta, Georgia, USA

– Herakleitos of Ephesus, c. 500 bce

“The viewer should see the object as I saw it, but with a lot of room for his own interpretation….The painting is like a very clear mirror which is barely visible but unmistakably there,” reflects Spanish painter Antonio López García (b. 1936) (*1*). “I am nostalgic for an art of our times in which a greater number of people can participate” (*1*). Part of a contemporary movement rooted in the traditions of 17th-century Spanish realism, López García, Claudio Bravo, Bernando Torrens, Francisco Roa, and many others have drawn inspiration from the meticulous work of Francisco de Zurbarán, Jusepe de Ribera, and Diego Velásquez to create their own naturalist style (*2*).

Rejecting abstraction, these artists create paintings from a broad range of subjects, choosing, much like their predecessors, a few or even 1 subject, which they lavish with extreme seriousness and attention, focusing on the “credible detail, that small touch of the familiar,” that has long been part of the repertory of Spanish painters (*3*). The characteristic high technical quality is not an end in itself, and the work reaches far beyond mere representation of nature.

In the United States, revival of traditional painting skills in the latter part of the 20th century is credited to professor Richard Lack, who in his essay On the Training of Painters (1969) envisioned atelier training in the contemporary art scene, “Indeed if Western Civilization wishes to retain the art of painting as a living part of its culture, this may be our last hope.” He founded the pioneering Atelier Lack in Minneapolis, “a small island of traditional art training surrounded by a sea of hostile opinion” (*4*). Rigorous technique, which had fallen by the wayside in modern times, addressed quality of drawing, color plausibility, truthfulness of light and shadow, highly developed skills of execution, and overall faithful depiction of nature.

The atelier attracted students from all over the world and became the model for similar schools, among them The New York Academy of Art and the Charles H. Cecil Studios in Florence, Italy. The New York Academy of Art won the support of Andy Warhol: “The course of art history,” he said, “would be changed if one thousand students could be taught Old Master drawing and painting techniques” (*5*).

In 1982, Lack coined the term “Classical Realism” because as he put it, “Any 20th century painting that suggests a recognizable object, however crudely or childishly rendered, qualifies as ‘realistic.’ Obviously, the simple word ‘realism,’ when applied to painting…is no longer meaningful” (*6*). But contemporary American realists are a disparate group, loosely characterized by a realistic approach to representation, which has persisted widely in the post-abstraction era.

A painter in the tradition of Spanish realism, Francisco Roa was born in Guadalajara, Spain, and moved to Madrid at age 18 to study at the Universidad Complutense and the Academia Peña. He has received considerable recognition at home, exhibiting in Madrid, Barcelona, and Granada. In 1993, he took his works to Lisbon and Miami, and a year later, to New York and Atlanta.

Extremely careful with detail, Roa, like many of his contemporaries, conveys an individual perception of reality, positioning everyday objects deliberately, proposing his own space and time. Sands Flowers, on this month’s cover, first exhibited in the United States in 1994, is a characteristic still life.

Against a vacant background vaguely reminiscent of sand, the artist composes a geometric ensemble to anchor his main object of interest, a glass vessel filled with natural elements past their prime. The board base, stacked on the left, breaks up the horizontal field. The metal circle, weathered and discolored, is slightly off center, the backdrop deliberately smudged. Inside the glass lie red petals, crinkled and lifeless, the detritus of beauty. Crammed in near the top is a hornet’s nest, and out of each side, dried blooms jut pathetically, their stems distorted and petals curled.

“All forms of beauty, like all possible phenomena,” wrote Charles Baudelaire in his On the Heroism of Modern Life, “contain an element of the eternal and an element of the transitory—of the absolute and the particular” (*7*). In a perfect balance of feeling and form, Roa’s poignant scene meets Baudelaire’s requirement. For nothing expresses the ephemeral better than flowers. Roses or sand verbenas, they perish all too soon, a metaphor for human transience and fragility.

The eternal and the absolute are elements artists have sought in the formalities of realism and the fragments of abstraction, on the same pathway, 1 step forward, 2 steps back; and scientists likewise, for rarely does excellent science or art occur without reference to past knowledge and principle.

In Sands Flowers, Roa probes the precariousness of existence, space and time, and life and death. His realistic representation of natural objects in decline provokes speculation—not only on the passage of time and inevitability of death, but for us, also on disease, which unduly hastens the process. In an unending circle, old scourges become new, among them tuberculosis, a hornet’s nest of multiple drug resistance, and now extensive resistance to second-line drugs, raising the specter of potentially untreatable disease. Roa sought essence in the staying power of exacting detail. In science too, on the same pathway, sometimes solutions lie simply in the core values: treatment standards, effective precautions, improved technology, better medicines, vaccines, and diagnostic tests (*8*).
